# Patient-specific static and dynamic 3D-printed models for planning endovascular repair of complex aortic disease with physician-modified stent grafts

**DOI:** 10.1186/s42155-026-00755-y

**Published:** 2026-08-15

**Authors:** Qincheng Gong, Yuanting Yang, Hongping Deng, Xiaoping Hu, Ji Zhang, Sheng Cao, Hongning Song, Qing Zhou

**Affiliations:** 1 Department of Ultrasound Imaging, Renmin Hospital of Wuhan University, No. 238 Jiefang Road, Wuchang District, Wuhan, Hubei Province 430060 China; 2 Department of Vascular Surgery, Renmin Hospital of Wuhan University, No. 238 Jiefang Road, Wuchang District, Wuhan, Hubei Province 430060 China; 3 Department of Cardiovascular Surgery, Renmin Hospital of Wuhan University, No.238 Jiefang Road, Wuchang District, Wuhan, Hubei Province 430060 China

**Keywords:** 3D printing, Complex aortic aneurysm, Endovascular repair, Surgical planning, Physician-modified stent grafts

## Abstract

**Objective:**

To develop a patient-specific static and dynamic 3D-printing workflow for preoperative planning of endovascular repair in complex aortic disease with physician-modified stent grafts, to assess the respective roles of rigid anatomical and dynamic models, and to explore its association with intraoperative efficiency compared with conventional image-based planning.

**Methods:**

This retrospective study included 46 consecutive patients with complex aortic disease encompassing various segments between the aortic arch and the abdominal aorta, who underwent endovascular repair with physician-modified stent grafts. Patients were assigned to a 3D printing-guided group (*n* = 22) or a conventional image-guided group (*n* = 24) based on the preoperative planning strategy. In the 3D printing group, patient-specific 1:1 rigid anatomical models and compliant dynamic models were generated from computed tomography angiography data. Rigid models were used for anatomical visualization, device sizing, and fenestration planning. Dynamic models, fabricated using silicone material (Shore hardness 10A) and integrated into a pulsatile flow system, were used to simulate device passage, deployment, and device–vessel interaction under physiological flow conditions. Model accuracy was evaluated by surface deviation analysis, and dynamic model performance was evaluated by Doppler-based flow comparison with in vivo measurements. The two groups were compared regarding operative time, radiation dose, contrast volume, perioperative complications, and short-term outcomes.

**Results:**

Rigid anatomical models demonstrated high geometric fidelity, with over 97% of surface deviations within 0.2 mm compared with CTA data. Dynamic models showed close agreement with in vivo hemodynamics, with comparable peak systolic velocities at the inlet (1.48 ± 0.20 m/s vs. 1.52 ± 0.18 m/s, *p* = 0.274), aneurysmal segment (1.20 ± 0.18 m/s vs. 1.25 ± 0.15 m/s, *p* = 0.312), and outlet (1.35 ± 0.17 m/s vs. 1.38 ± 0.16 m/s, *p* = 0.401). In the 3D printing-guided group, simulation findings were concordant with intraoperative findings in 21 of 22 cases. Compared with rigid static models, dynamic models provided additional planning information in anatomically challenging cases, including severe angulation of arch or neck, severe luminal stenosis or true lumen collapse, target vessels arise from the aneurysm sac, complex target vessel proximal segment and severe access tortuosity. In the exploratory clinical comparison, the 3D-printing-guided group had significantly shorter operative time (133.6 ± 43.3 min vs. 171.4 ± 72.1 min, *p* = 0.039) and lower contrast volume (181.7 ± 68.6 mL vs. 234.9 ± 101.0 mL, *p* = 0.044) than the conventional image-guided group. The need for intraoperative device adjustment did not differ significantly between groups. No major adverse events occurred within 30 days in either group.

**Conclusion:**

A patient-specific dual-model 3D printing workflow combining rigid anatomical models and dynamic models is feasible for planning endovascular repair of complex aortic disease with physician-modified stent grafts. The dynamic model provides complementary information beyond static anatomical assessment, particularly in anatomically complex cases requiring simulation of device–vessel interaction. The observed reductions in operative time and contrast use suggest potential intraoperative benefits.

**Supplementary Information:**

The online version contains supplementary material available at 10.1186/s42155-026-00755-y.

## Manuscript

### Introduction

Complex aortic disease remains technically challenging for endovascular repair, particularly in elderly patients and those with hostile anatomy [1, 2]. These lesions are often associated with severe vascular tortuosity, angulated or short landing zones, branch-vessel involvement, and unfavorable luminal morphology, all of which may complicate device delivery, fenestration alignment, and sealing. Although endovascular repair has reduced perioperative risk compared with open surgery [3], procedural planning and execution in such anatomically complex settings remain demanding, with persistent risks of suboptimal stent graft apposition, intraoperative adjustment, endoleak, and branch-related complications [4].

In these situations, fenestrated or branched endovascular strategies are often required to preserve branch perfusion while achieving adequate exclusion of the diseased aortic segment. Commercially manufactured custom-made devices may provide an effective option in selected patients; however, their routine use may be limited by availability, regulatory and reimbursement constraints, and prolonged manufacturing lead times. During the study period, custom-made fenestrated or multi-branched devices were not routinely used in our setting. Therefore, physician-modified stent grafts were adopted as a practical alternative after multidisciplinary evaluation, particularly when timely treatment or individualized device configuration was required.

Accurate preoperative planning is essential for endovascular repair with physician-modified stent grafts. Conventional planning is primarily based on computed tomography angiography (CTA), three-dimensional workstation reconstruction and costly preoperative planning software. Although these tools provide detailed anatomical information, they remain limited in their ability to reproduce tactile anatomy, vascular compliance, pulsatile deformation, device-vessel alignment during stent graft modification and device-vessel interaction during stent graft passage and deployment. These limitations may be especially relevant in complex aortic disease with marked tortuosity, challenging landing zones, dissecting morphology with narrow true lumen, or branch vessels arising from aneurysmal segments.

Three-dimensional (3D) printing has emerged as a promising adjunct to CTA-based planning by enabling the creation of patient-specific vascular models derived from imaging data [5–8]. In routine clinical practice, however, most 3D-printed vascular models are rigid and therefore mainly provide static anatomical information. While such models are valuable for anatomical visualization, device sizing, and fenestration planning, they cannot reproduce vascular compliance or dynamic device-vessel interaction. To address these limitations, we developed a patient-specific dual-model 3D-printing workflow consisting of rigid anatomical models and compliant dynamic flow models connected to a pulsatile flow system. The rigid models were used for geometric and morphological assessment, whereas the dynamic models were used to simulate device passage, deployment behavior, and stent-graft-vessel interaction under near-physiological flow conditions. We hypothesized that these two model types would play complementary roles in procedural planning, with dynamic simulation providing additional information beyond static anatomical assessment alone.

Accordingly, the primary aim of this study was to develop a patient-specific static and dynamic 3D-printing workflow for planning endovascular repair of complex aortic disease with physician-modified stent grafts, and to compare the complementary roles of rigid and compliant models in preoperative planning and simulation. As a secondary exploratory analysis, we also compared perioperative and short-term follow-up outcomes between patients treated using a 3D-printing-guided planning workflow and those treated using conventional CTA-based image guidance alone.

## Materials and methods

### Study population

This retrospective comparative study included 46 consecutive patients with complex aortic disease who underwent endovascular repair of complex aortic disease with physician-modified stent grafts at Renmin Hospital of Wuhan University between March 2021 and March 2025. The lesions involved various segments between the aortic arch and the abdominal aorta. Complex aortic disease was defined as aortic pathology with anatomical features that made standard endovascular repair technically challenging, including visceral or supra-aortic branch involvement, a proximal landing zone < 15 mm, neck angulation > 60°, severe tortuosity, unfavorable branch orientation, or a narrow true lumen.

The inclusion criteria were (1) anatomical or clinical high-risk factors for open repair; (2) the need for branch preservation by endovascular repair with physician-modified stent grafts; and (3) availability of complete preoperative CTA and perioperative data. The exclusion criteria were contraindications to endovascular repair, active infection, major thrombotic disease, previous open aortic surgery, or incomplete imaging or follow-up data.

Patients were grouped according to the preoperative planning strategy into a 3D-printing-guided group (*n* = 22) and a conventional image-guided group (*n* = 24). In the 3D-printing-guided group, the decision to utilize the 3D-printing workflow was made by the treating team based on a comprehensive assessment of anatomical complexity, the anticipated difficulty of fenestration alignment, the expected clinical value of deployment simulation, patient preference, and surgical urgency.

The study protocol was approved by the institutional ethics committee (WDRY2020-K230), which waived the requirement for informed consent.

### CTA raw data acquisition

Patient-specific computed tomography angiography (CTA) images of the aorta were acquired using a 256-slice GE Revolution CT scanner (GE Healthcare, USA) with a slice thickness of 0.625 mm. Images were reconstructed on a GE ADW 4.6 workstation and exported in Digital Imaging and Communications in Medicine (DICOM) format for further segmentation and model generation.

#### 3D printing of anatomical models

**Fig. 1:**
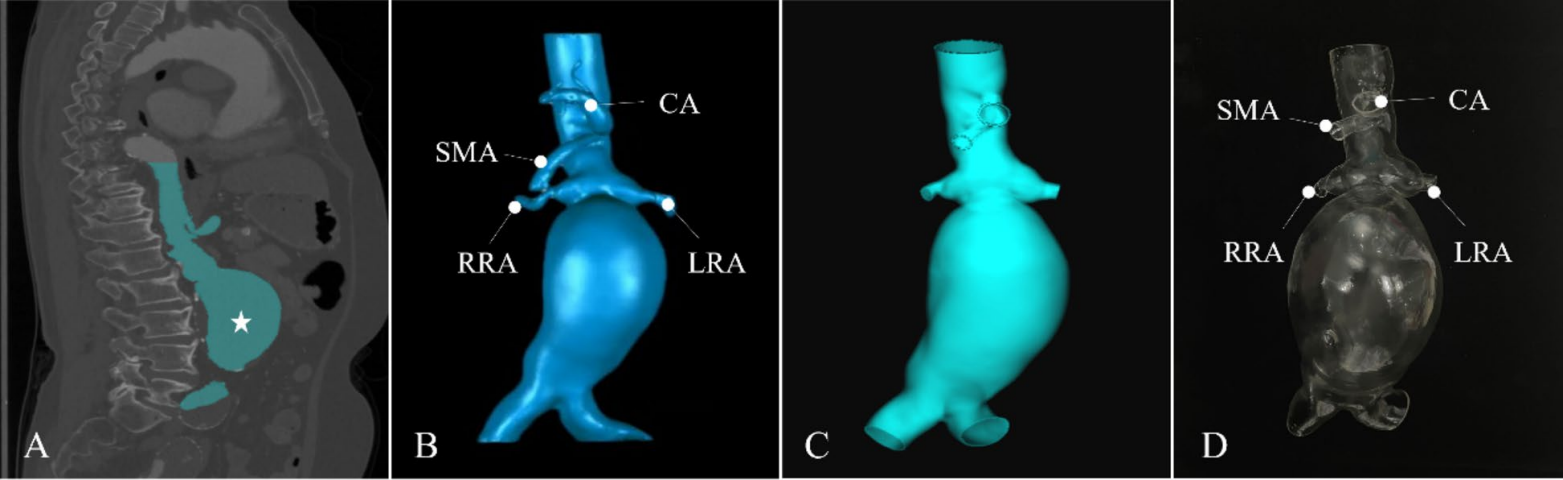
Workflow for 3D printing a model of abdominal aortic aneurysm (AAA). **A** CTA scan showing the abdominal aortic aneurysm (AAA) (★), with the aneurysmal blood pool highlighted in the original image. **B** 3D reconstruction of the AAA blood pool. **C** Generation of the AAA wall model by expanding the blood pool mask by 1 mm to account for wall thickness, followed by subtraction of the original blood pool mask to isolate the aneurysm wall structure. **D** Final AAA 3D model printed directly using resin material. CT, celiac artery; SMA, superior mesenteric artery; RRA, right renal artery; LRA, left renal artery

DICOM data were imported into Mimics (Materialise, Leuven, Belgium) for segmentation and three-dimensional reconstruction. The blood pool was segmented using threshold-based isolation followed by region-of-interest cropping. A vascular wall model was then created in 3-matic (Materialise, Leuven, Belgium) by outward expansion of the blood-pool model by 1 mm and Boolean subtraction [9]. The final model was exported as a stereolithography (STL) file and printed at a 1:1 scale using rigid resin (Fig. 1).

#### 3D printing of dynamic functional models

**Fig. 2:**
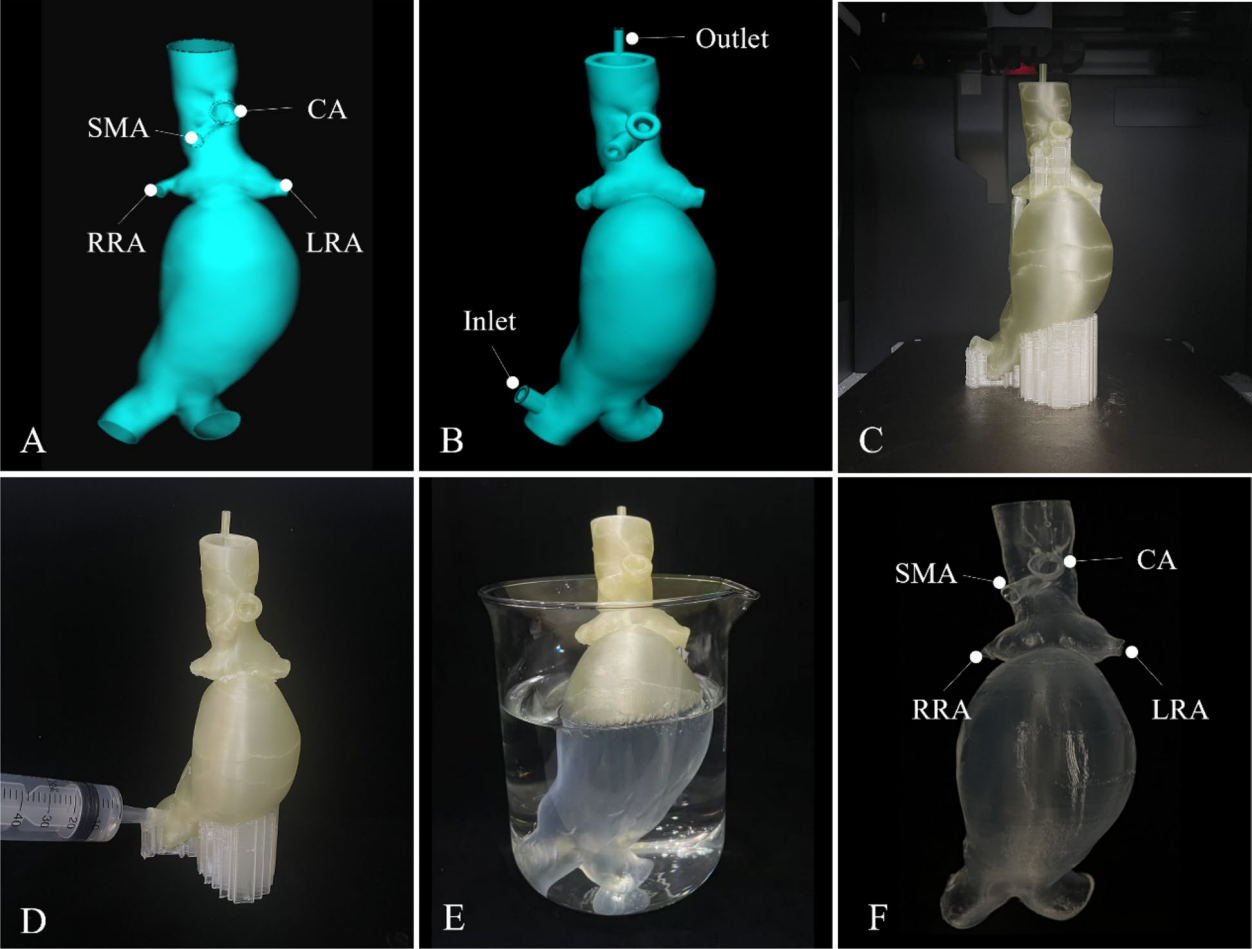
Workflow for 3D printing a flexible model of abdominal aortic aneurysm (AAA) using a mold-modelling method. **A** Digital model of the AAA wall. **B** Generation of the AAA wall mould by expanding the original 3D wall model by 2 mm and subtracting it to create a mould cavity; the mould was printed using water-soluble PVA. **C** 3D printing of the AAA wall mould with water-soluble PVA material. **D** Injection of silicone into the mould using a syringe. **E** Partial dissolution of the mould in water: the upper portion remains intact, while the lower portion has dissolved, revealing the inner silicone model. **F** The fully formed silicone AAA model after complete removal of the PVA mould

Dynamic models were constructed as patient-specific vascular phantoms integrated into a mock circulatory system [10]. These models were designed to simulate device passage, deployment, and device-vessel interaction under pulsatile flow conditions. The dynamic models were fabricated using a mold-based casting technique. Briefly, a water-soluble polyvinyl alcohol (PVA) mold was printed from the STL file, filled with silicone material, cured, and then dissolved in water to obtain a flexible vascular model (Fig. 2).

The fabrication time was defined as the entire process from CTA acquisition to completion of the final sterilized model.

#### Material selection

Based on our group’s previous studies [11], materials used for model fabrication included transparent photopolymer resin TangoPlus (FLX930, Stratasys, USA) and three types of silicone (Silicone 5A, 10A, and 20A; Hong Cheng Silicone Products, Guangdong, China). Their mechanical properties—including elastic modulus and tensile strength—were evaluated using a universal testing machine (CMT-6203, MTS, USA) and a Shore A hardness tester. Each material was used to construct a prototype of a large vessel model, which was integrated into a pulsatile flow loop. The pump cycle was set at 0.8 s (systolic: 0.3 s; diastolic: 0.5 s), simulating a heart rate of 75 beats per minute. The optimal material was selected based on performance in both static and dynamic flow conditions. Based on these results, Silicone 10A was selected for fabrication of the final dynamic models.

#### Hemodynamic simulation

**Fig. 3:**
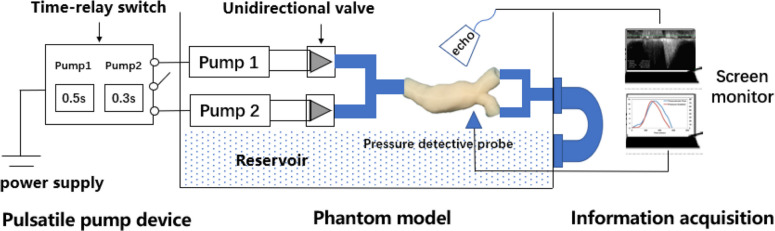
Mock circulatory system for hemodynamic simulation of abdominal aortic aneurysm (AAA). **A** Pulsatile pump device: Two pumps controlled by a time-relay switch generate pulsatile flow through unidirectional valves. **B** Phantom model: A 3D-printed soft AAA model connected in a closed-loop circuit with embedded pressure sensor. **C** Information acquisition: Flow and pressure data are collected via ultrasound and displayed on a screen for analysis

A dual-pump pulsatile flow loop was designed to simulate physiological hemodynamics. The system included two centrifugal pumps (SUNSUN HJ-6000 [150 W] and HJ-4500 [80 W]; SUNSUN Group Co., Ltd., Zhejiang, China), controlled by a programmable time-relay switch to alternate systolic and diastolic phases. Each pump was equipped with a unidirectional valve to prevent retrograde flow. The pumps were connected to the flexible 3D-printed model via a T-shaped connector to form a closed-loop system. The working fluid consisted of distilled water mixed with 30% glycerol and 0.1% starch to approximate the viscosity and rheological properties of blood. Ultrasound scattering particles (5 µm) were added to enhance Doppler signal quality [12]. Color Doppler ultrasound was used to visualize flow patterns, while spectral Doppler was employed to measure peak systolic velocities (PSVs) at the inlet, aneurysmal, and outlet segments of the model (Fig. 3).

### Sterilization and accuracy assessment of 3DP models

Before simulation, the models were sterilized using ethylene oxide for 8 h. The models were first rescanned using the aforementioned CT equipment; subsequently, their dimensional accuracy was assessed by registering the acquired scan data to the source CTA-derived digital models, followed by geometric comparison included in 3-matic. The analytical procedures included wall thickness analysis and part comparison analysis, which were performed to assess the thickness uniformity of the fabricated models and their geometric consistency with the CTA-derived digital models, respectively.

### Surgical simulation with 3DP models

In the 3DP-guided group, preoperative simulations were performed by two vascular surgeons using patient-specific rigid and dynamic models. The rigid models were used for device sizing, alignment, and fenestration positioning, whereas the dynamic models were used to assess stent graft deployment, device-vessel interaction, and the risk of endoleak under pulsatile flow conditions. Following simulated stent graft deployment in the dynamic model, contrast medium was introduced into the flow loop. A positive endoleak prediction was defined as visible contrast leakage outside the stent graft into the simulated aneurysm sac or clearly suboptimal stent graft apposition, whereas a negative prediction was defined as complete exclusion of the aneurysm sac without visible contrast leakage. Final surgical plans were determined by consensus (Supplementary Movie 1).

In the conventional image-guided group, CTA data were imported into semi-automatic software (Endosize; Therenva, Rennes, France) for 3D reconstruction and vessel centerline generation. Subsequently, key parameters were measured, including aortic diameters at the proximal and distal landing zones and at each branch level, as well as the diameters, centerline distances, and ostial orientations of the branch arteries. These measurements were used to determine stent graft sizing and the intraoperative fenestration sites on the stent grafts, without using 3D-printed models.

**Fig. 4:**
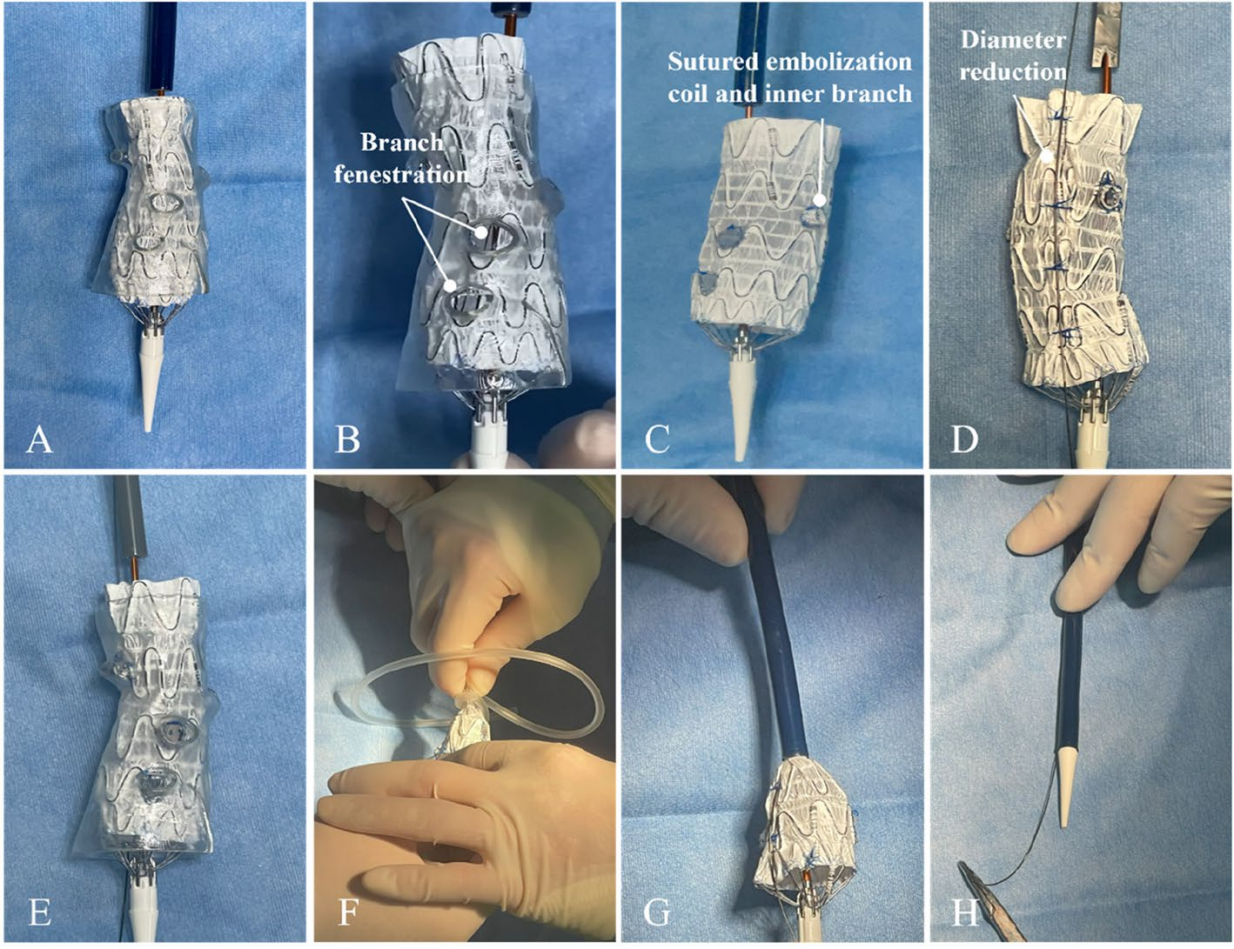
Procedural simulation workflow based on 3D-printed models. **A** Deployment of the vascular stent graft into the 3D-printed model. **B** Fenestration of the stent graft based on the corresponding branch vessels. **C** Suturing of embolization coil and inner branches. **D** Completion of diameter reduction after fenestration and inner branch attachment. **E** Repositioning of the modified stent graft in the model to confirm alignment. **F**–**H** Retrieval of the personalized stent graft into the delivery sheath, ready for in vivo deployment

For both groups, the stent graft modification procedure comprised the following steps: marking the target sites, creating the fenestrations, suturing the coils and inner branches, applying diameter-reducing ties, and resheathing the device (Fig. 4).

All endovascular procedures were performed in the same hybrid operating room equipped with a fixed imaging system. Standard fluoroscopy and digital subtraction angiography were used in both groups. Fusion imaging and Fiber Optic RealShape technology were not used. During the procedure, intraoperative adjustments, device selection, operating time, and technical success were recorded.

## Statistical analysis

Continuous variables are presented as mean ± standard deviation (SD), and categorical variables as number (percentage). Comparisons of continuous variables between the 3DP and conventional groups were performed using the independent-samples *t*-test or Mann–Whitney *U* test, as appropriate. Categorical variables were compared using Pearson’s chi-square test or Fisher’s exact test. All statistical analyses were two-tailed, and a *p*-value < 0.050 was considered statistically significant.

## Results

### Patient characteristics

A total of 46 patients were included, with 24 in the conventional image-guided group and 22 in the 3D printing-guided group. The mean age was 68.9 ± 8.8 years, and 67.3% were male. The most common pathology was abdominal aortic aneurysm (56.5%), followed by thoracic aortic aneurysm (26.1%) and thoracoabdominal aortic aneurysm (17.4%). Elective surgery was performed in 82.6% of patients, and 17.4% were treated urgently. Anatomical complexity features included chronic dissection (21.7%), prior type I endoleak repair (10.9%), aneurysm diameter ≥ 55 mm (39.1%), neck angulation > 60° (17.4%), and severe calcification (30.4%). There were no significant differences in baseline demographic, clinical, or anatomical characteristics between the two groups (Table 1, all *p* > 0.050).

**Table 1:** Baseline characteristics of patients in the 3DP-guided and image-guided groups

Variables	Total (*n* = 46)	3DP-guided group (*n* = 22)	Image-guided group (*n* = 24)	*p*-value
Clinical variables				
Age, years	68.9 ± 8.8	68.0 ± 9.8	69.3 ± 8.3	0.711
Male sex, *n* (%)	31 (67.3%)	15 (68.2%)	16 (66.7%)	0.913
History of smoking, *n* (%)	17 (40.0%)	7 (31.8%)	10 (41.7%)	0.489
Coronary artery disease, *n* (%)	20 (43.5%)	9 (40.9%)	11 (45.8%)	0.736
Diabetes mellitus, *n* (%)	9 (19.6%)	4 (18.2%)	5 (20.8%)	> 0.999
Hypertension, *n* (%)	35 (76.1%)	16 (72.7%)	19 (79.1%)	0.609
COPD, *n* (%)	7 (15.2%)	4 (18.2%)	3 (12.5%)	0.900
ASA class IV, *n* (%)	22 (47.8%)	10 (45.4%)	12 (50.0%)	0.768
Type of aortic aneurysm				
Abdominal aortic aneurysm (AAA), *n* (%)	26 (56.5%)	13 (59.0%)	13 (54.2%)	0.736
Juxtarenal AAA	21 (41.3%)	9 (40.9%)	10 (41.7%)	0.958
Pararenal AAA	7 (15.2%)	4 (18.2%)	3 (12.5%)	0.900
Thoracoabdominal aortic aneurysm (TAAA), *n* (%)	8 (17.4%)	3 (13.6%)	5 (20.8%)	0.800
Thoracic aortic aneurysms (TAA), *n* (%)	12 (26.1%)	6 (27.2%)	6 (25.0%)	0.861
Procedural urgency				
Elective/Semi-urgent, *n* (%)	38(82.6%)	19(86.3%)	19(79.2%)	0.800
Urgent, *n* (%)	8(17.4%)	3(13.6%)	5(20.8%)	0.800
Anatomical characteristic				
Chronic aortic dissection	10 (21.7%)	4 (18.2%)	6 (25.0%)	0.840
Type I endoleak repair	5 (10.9%)	3 (13.6%)	2 (8.3%)	0.918
Aneurysm diameter ≥ 55 mm, *n* (%)	18 (39.1%)	8 (36.4%)	10 (41.7%)	0.713
High aneurysm neck angle (> 60°), *n* (%)	8 (17.4%)	4 (18.2%)	4 (16.7%)	> 0.999
Severe vessel calcification, *n* (%)	14(30.4%)	6 (26.7%)	8 (33.3%)	0.655

*ASA* American Society of Anesthesiologists, *COPD* chronic obstructive pulmonary disease

Values are presented as mean ± standard deviation or number (percentage). *P*-values were calculated using Student’s *t*-test or chi-square test where appropriate

### Evaluation of 3D-printed models

#### Anatomical accuracy of 3D-printed models

**Fig. 5:**
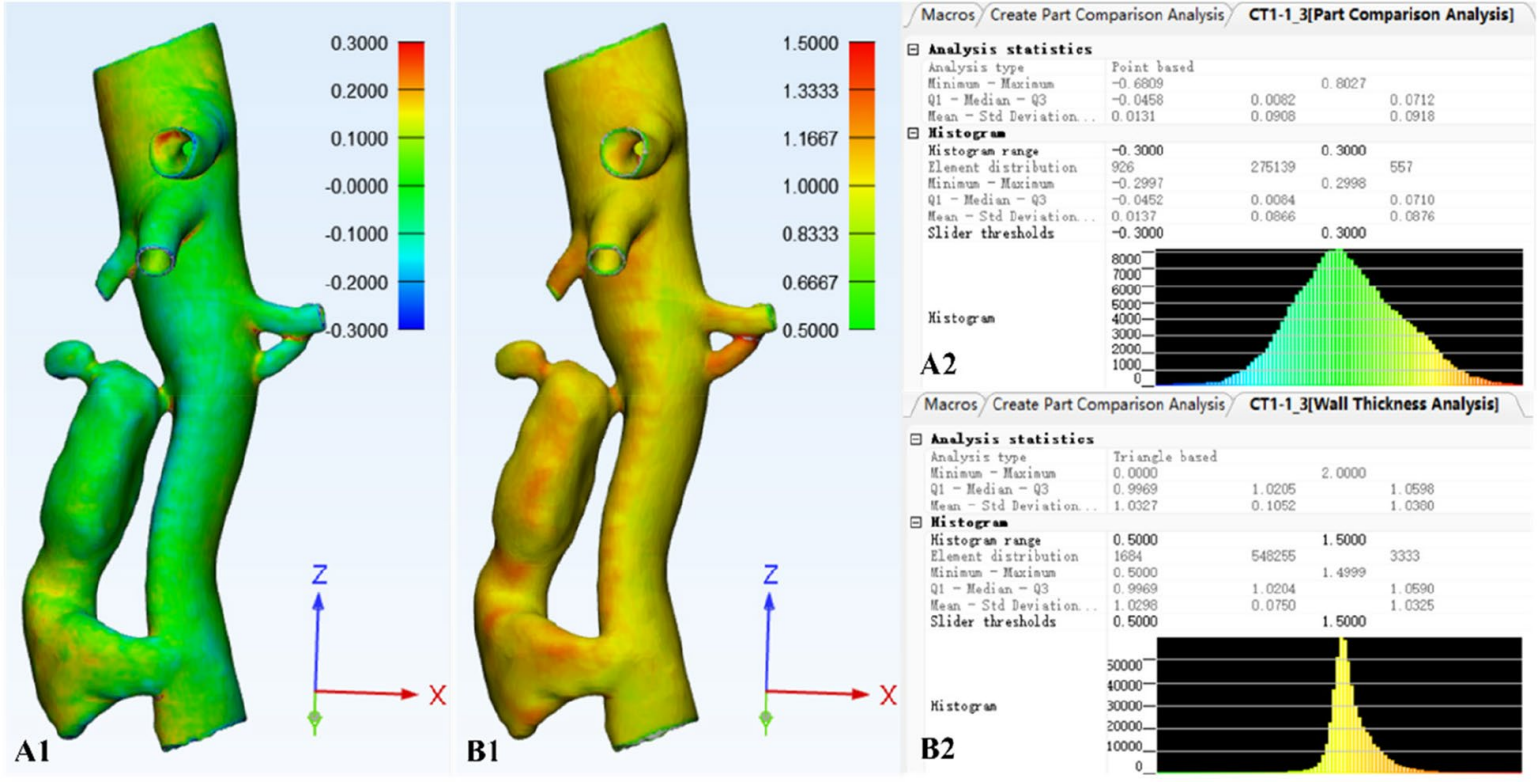
Assessment of anatomical accuracy of 3D-printed abdominal aortic aneurysm (AAA) models. **A1** Part comparison analysis colormap and **A2** histogram showing the distribution of surface deviation distances between the 3D-printed vascular model and the digital model. **B1** Wall thickness analysis colormap and **B2** histogram showing the wall thickness distribution of the 3D-printed vascular wall model

Quantitative comparison between the printed aortic models and the source CTA-based digital models demonstrated high anatomical accuracy. Wall thickness analysis showed a mean model thickness of 1.03 mm with a homogeneous distribution on the colormap. In part comparison analysis, the mean surface deviation was 0.01 mm, with 74% of surface points showing a deviation of < 0.1 mm and 97% showing a deviation of < 0.2 mm. Deviation mapping showed that localized discrepancies were mainly observed in regions where the branch-vessel takeoff angle relative to the aorta was small. These results support the geometric fidelity and reproducibility of the printed anatomical models (Fig. 5).

#### Hemodynamic performance evaluation of dynamic models

##### Material property assessment

Mechanical testing of TangoPlus and three silicone materials (5A, 10A, and 20A) showed that silicone 10A most closely approximated the mechanical properties of native aortic tissue in terms of elastic modulus and tensile strength [13] (Table 2). Under physiological pulsatile conditions, silicone 10A maintained structural integrity, whereas silicone 5A showed excessive compliance and silicone 20A failed because of excessive stiffness.

**Table 2:** Mechanical properties and dynamic testing of materials

	Mechanical properties			Hemodynamic test		
Material	Elastic modulus (E, MPa)	Tensile strength (Rm, MPa)	Shore A hardness	Deformation degree	Pressure toleration	Pressure maintainance
Silicone 5	0.1	2.5	0–3	Large	Good, no rupture	70–80/40–45 mmHg
Silicone 10	0.9	3.0	9–11	Good	Good, no rupture	Normal pressure (120/80 mmHg)
Silicone 20	2.1	3.5	19–21	Least	Rupture	Normal pressure (120/80 mmHg)
TangoPlus	2.5	1.5	24–26	Not tested	Not tested	Not applicable
Native AAA wall	1.2*	2.5*	10–12*	Normal	Normal	Normal pressure (120/80 mmHg)

* denotes values cited from reference [12, 14]

##### Dynamic results evaluation

**Fig. 6:**
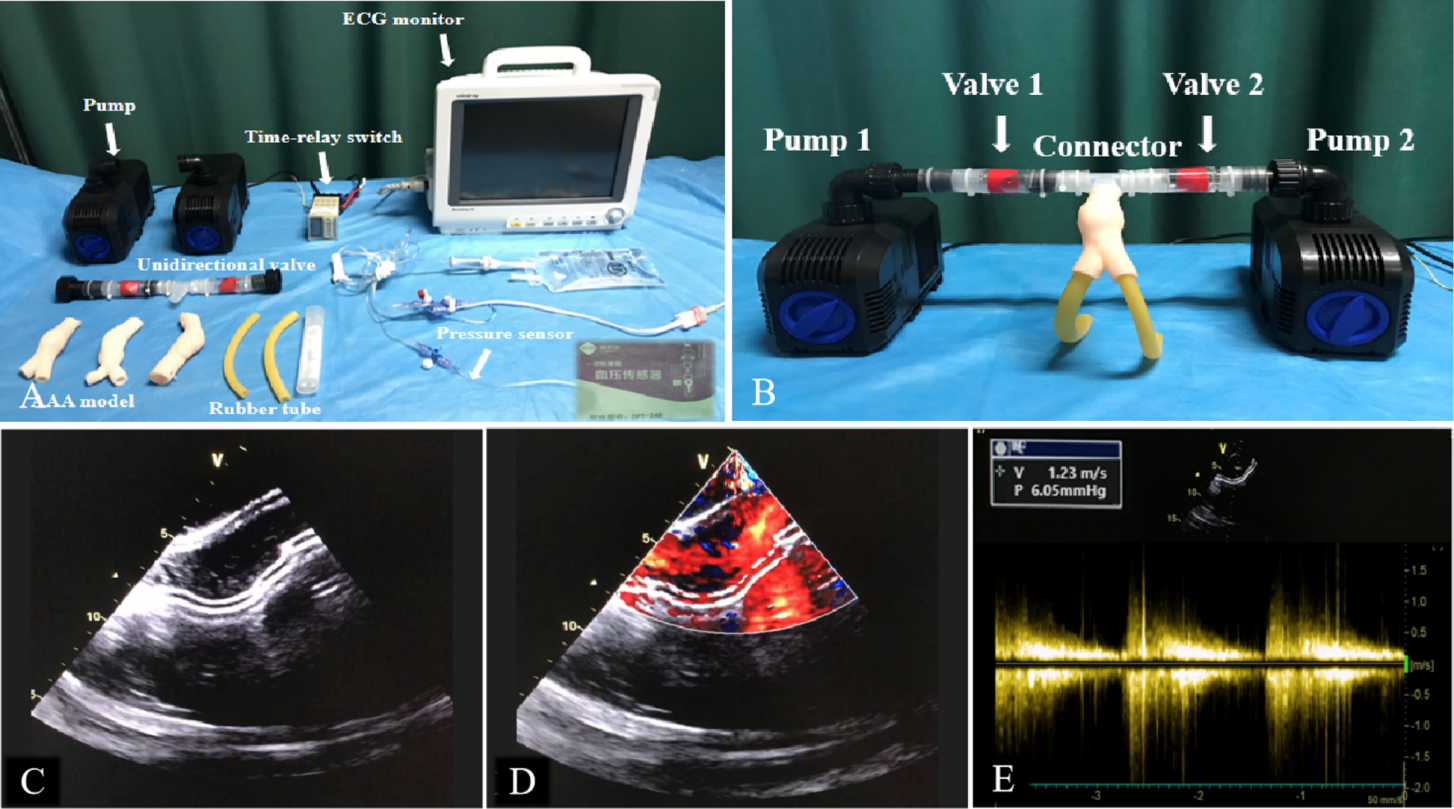
Hemodynamic simulation and ultrasonic flow acquisition in the AAA model. **A**–**B** Two pumps with unidirectional valves and connector with the phantom AAA model. **C** Two-dimensional ultrasound to observe and measure AAA morphology. **D** Color Doppler to demonstrate intraluminal blood flow pattern: the inlet and outlet of AAA show the red or colored blood flow, the blood flow in the tumor cavity is dim, showing red and blue blood flow. **E** Spectral Doppler to detect the peak systolic velocity (PSV)

Dynamic testing showed that the 3D-printed models closely approximated in vivo hemodynamic conditions. The measured peak systolic velocities were 1.48 ± 0.20 m/s at the inlet, 1.20 ± 0.18 m/s within the aneurysmal segment, and 1.35 ± 0.17 m/s at the outlet, with no significant differences compared with the corresponding clinical Doppler measurements (1.52 ± 0.18 m/s, 1.25 ± 0.15 m/s, and 1.38 ± 0.16 m/s, respectively; all *p* > 0.050). Flow velocity within the aneurysmal cavity was consistently lower than that at the inlet and outlet, and turbulent flow patterns were frequently observed. More pronounced turbulence and flow separation were observed in larger aneurysms (Table 3, Fig. 6).

**Table 3:** Hemodynamic results in patient-specific models

Parameter	In vivo	3DP model	*p*-value
Inlet velocity (m/s)	1.52 ± 0.18	1.48 ± 0.20	0.274
Aneurysmal velocity (m/s)	1.25 ± 0.15	1.20 ± 0.18	0.312
Outlet velocity (m/s)	1.38 ± 0.16	1.35 ± 0.17	0.401

#### Fabrication time analysis

**Fig. 7:**
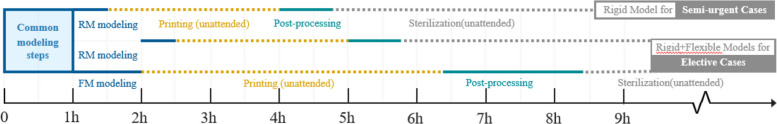
Single-operator 3D printing workflow for abdominal aortic models under varying clinical urgencies. RM, rigid model; FM, flexible model

All models were completed as scheduled within the planned preoperative timeframe. The mean fabrication time was 14.2 ± 1.2 h for rigid anatomical models and 19.3 ± 3.6 h for dynamic functional models (Table 5). The longer fabrication time for dynamic models reflected the additional steps required for material preparation, assembly, and functional testing. When rigid and dynamic models were manufactured concurrently, the total fabrication time was comparable to that required for the dynamic model alone, as the rigid model could be produced during the same workflow without extending the overall timeline (Fig. 7).

### Surgical simulation and clinical application

#### Surgical simulation in the 3DP-guided group

In 22 patients with complex aneurysms, patient-specific 3D-printed models were used for preoperative simulation (Table 4). The simulations correctly predicted stent sizing, fenestration alignment, deployment strategy, and endoleak risk in 21 of 22 cases (95.5%). One intraoperative death occurred because of aneurysm rupture and was considered unrelated to model guidance.

**Table 4:** PMSG modification simulation results based on 3D-printed anatomic and dynamic functional models (*n* = 22)

Pt	Age	Sex	Type of aneurysm	Max diameter (mm)	Stent type and size	Involved branches and treatment	Predicted risk of endoleak	Added value of dynamic model (Y/N)	Agreement with actual surgery (Y/N)
1	75	M	TAA	61	Ankura 46 × 200	F (IA, LCCA, LSA)	No	Y/1	Y
2	70	M	TAAA(AD)	52	Ankura 46 × 200	F (LCCA、LSA)	No	Y/2	Y
3	50	M	TAAA(AD)	54	Ankura 30 × 26 × 160	F (CA, SMA, LRA, RRA)	No	Y/2 + 4	Y
4	79	M	J-AAA	75	Yuranos 28 × 12 × 120	F(RRA)	Yes	N	Y
5	74	M	J-AAA	35	Yuranos 24 × 12 × 120	F (LRA)	Yes	N	Y
6	72	M	J-AAA	35	Endurant 23 × 13 × 170	F (RRA)	Yes	N	Y
7	67	M	J-AAA	49	Yuranos 28 × 12 × 120	F (LRA)	No	N	Y
8	64	F	TA(ER)	58	Ankura 34 × 200	F (IA, LCCA, LSA)	Yes	Y/1	Y
9	43	M	TAAA(AD)	48	Ankura 32 × 28 × 120	F (CA, SMA, LRA, RRA)	Yes	Y/2 + 3 + 4	Y
10	68	F	TAA	59	Valiant 30 × 200	E(LSA)	Yes	Y/1 + 3	Y
11	71	M	TAA	44	Castor 34 × 28 × 200	F(LSA) + B(LCCA)	No	N	Y
12	76	M	AA(ER)	57	Ankura 28 × 28 × 80	F (CA, SMA, LRA, RRA)	Yes	N	Y
13	79	M	AA(ER)	69	None	F (CA, SMA, LRA, RRA)	Yes	Y/1	N
14	69	F	J-AAA	65	Yuranos 28 × 12 × 120	F (LRA, RRA)	Yes	Y/1 + 4	Y
15	67	M	TAA	57	Ankura 38 × 30 × 200	F (IA, LCCA, LSA)	Yes	Y/3 + 4	Y
16	74	M	P-AAA	45	Ankura 24 × 20 × 120	F (CA, SMA, LRA, RRA)	Yes	N	Y
17	72	M	P-AAA	47	Endurant 25 × 13 × 145	F (LRA, RRA)	No	Y/4	Y
18	83	M	J-AAA	42	Endurant 23 × 13 × 166	F (LRA)	No	N	Y
19	63	M	TAA	43	Ankura 36 × 32 × 160	F (RSA, LSA)	Yes	N	Y
20	65	M	J-AAA	52	Endurant 28 × 16 × 166	F (LRA, RRA)	Yes	N	Y
21	60	M	P-AAA	87	Endurant 28 × 16 × 166	F (SMA)	Yes	Y/1 + 3	Y
22	58	M	J-AAA	51	Ankura 28 × 24 × 120	F (CA, SMA, LRA, RRA)	Yes	N	Y

*AAA* abdominal aortic aneurysm, *TAA* thoracic aortic aneurysms, *TAAA* thoracoabdominal aortic aneurysm, *ER* endoleak repair, *AD* aneurysms with dissection, *CA* coeliac artery, *SMA* superior mesenteric artery, *RRA* right renal artery, *LRA* left renal artery, *LSA* left subclavian artery, *IA* innominate artery, *LCCA* left common carotid artery

Clinical application of the dynamic model was categorized as follows: Y/1, cases with severe angulation of arch or neck; Y/2, cases with true lumen collapse or severe luminal stenosis; Y/3, cases with target vessels arise from the aneurysm sac; Y/4, cases with complex target vessel ostia

Involved Branches and Treatment were categorized as follows: F—fenestration; B—branch; E—embolism

**Fig. 8:**
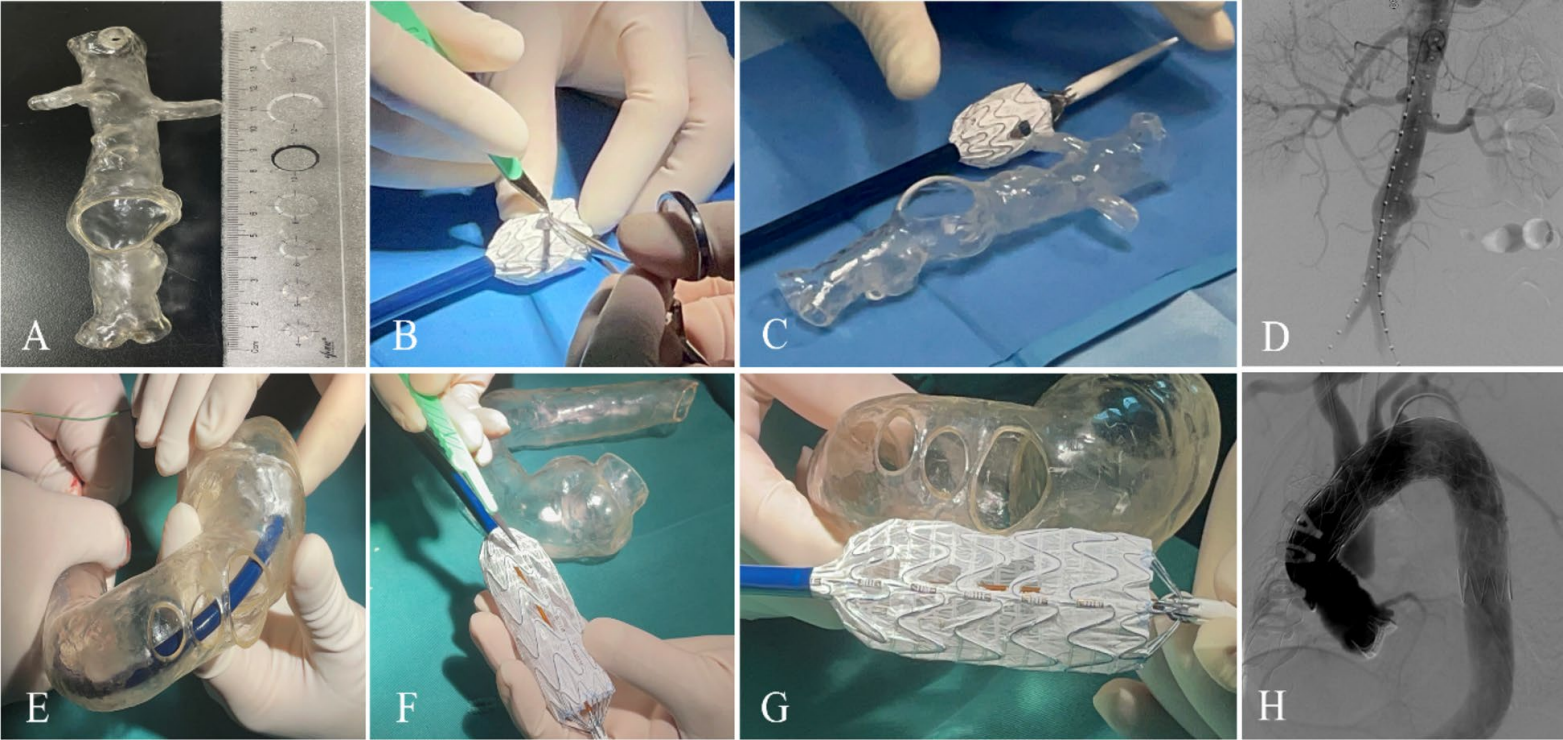
PMSG modification simulation using patient-specific rigid anatomical models. **A**–**D** Pt 5: Abdominal aortic aneurysm. Yuranos 24 × 12 × 120 mm stent graft covering the LRA; one fenestration created based on the 3D-printed model. Final intraoperative angiogram showing the implantation result. **E**–**H** Pt 8: Thoracic aorta endoleak repair. Ankura 34 × 200 mm stent graft covering the IA, LCCA, and LSA; three fenestrations guided by the model. Final intraoperative angiogram showing the implantation result

**Fig. 9:**
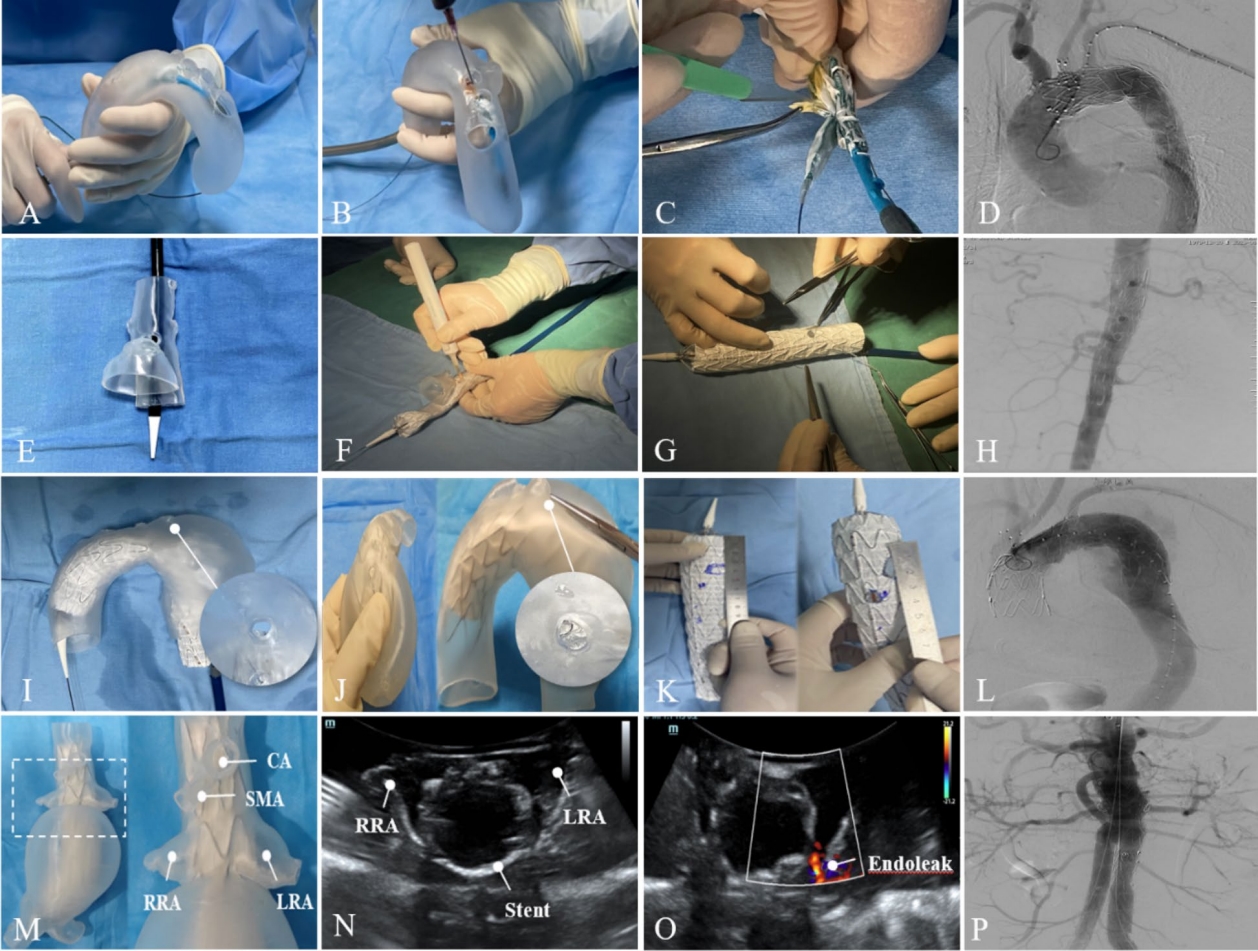
PMSG modification simulation using patient-specific flexible models. **A**–**D** Pt 11: Aortic arch aneurysm. Castor branched stent graft. The soft model allowed the stent to be more easily released and removed from the device with a lower risk of damage. **E**–**H** Pt 9: TAAA with dissection, with CA arising from the false lumen. Stent graft with four fenestrations (LRA, RRA, SMA, CA). The flexible model enabled safer and more compliant deployment simulation. **I**–**L** Pt 15: Aortic arch aneurysm, with LSA arising from the aneurysm sac. Stent graft with three fenestrations (IA, LCCA, LSA). The rigid model could not accommodate wall deformation well; the flexible model enabled manual compression for accurate alignment. **M**–**P** Pt 14: Abdominal aortic aneurysm. The model showed significant neck angulation and flared renal artery ostia. The flexible model, connected to a mock circulation system, revealed a localized type I endoleak, prompting strategy adjustment

Comparison between rigid and flexible models in 21 patients revealed that 47.6% (10/21) of cases showed equivalent results for fenestration planning (Fig. 8). In 52.4% (11/21), the flexible model demonstrated distinct advantages (Fig. 9). First, in six cases with severe angulation of arch or neck, rigid model could not predict device conformability in steep angulations. The flexible model was essential to physically simulate whether the device will adapt to the inner curve while avoiding damage to device. Second, in three dissecting aneurysms, rigid models failed to reproduce the narrow true lumen, resulting in difficulty in releasing and retrieving the device from the model and reduced agreement with intraoperative deployment. Third, in four cases in which the planned fenestrations corresponded to target vessel ostia located within an aneurysmal or dilated aortic segment, rigid models showed poor stent graft apposition, whereas flexible models facilitated the visualization and precise marking of fenestration sites. Fourth, in five cases with complex target vessel ostia, These models can visualize the anatomical trajectory, adjacent relationships, angulation and tortuosity of the target vessels. Additionally, the models can be directly trimmed to serve as templates for fenestration.

**Fig. 10:**
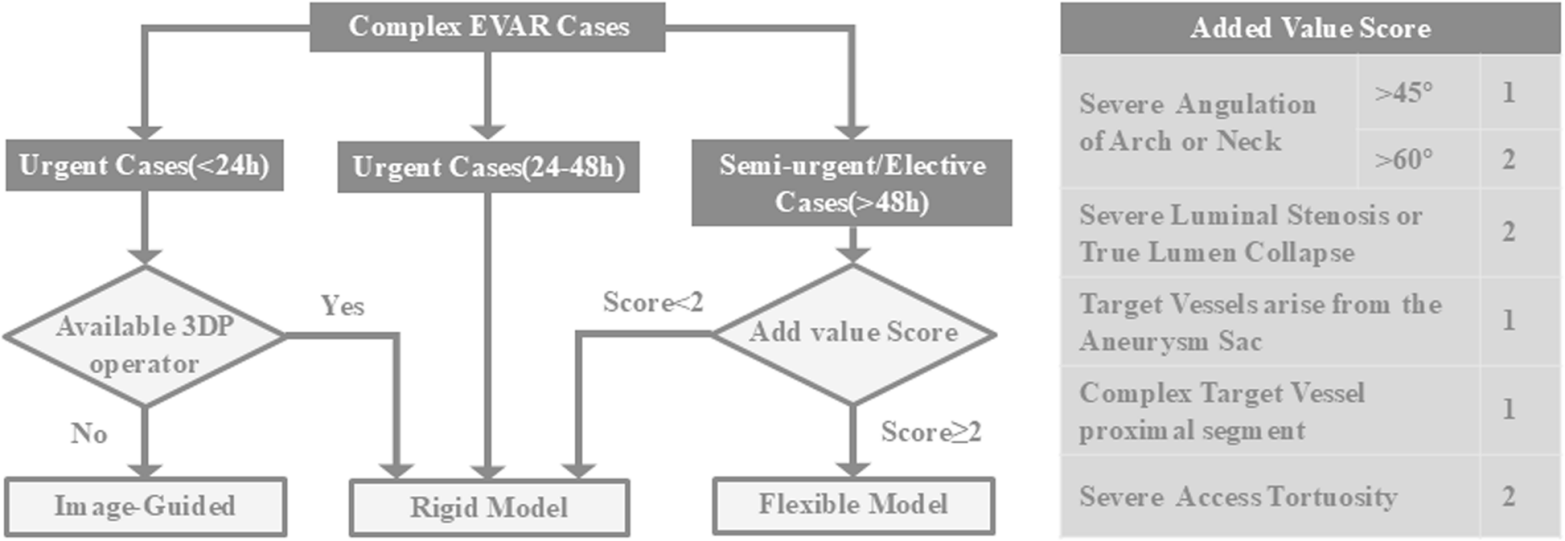
Flowchart for selecting rigid versus flexible 3D-printed models

Based on these clinical experiences, we developed a practical flowchart to guide the selection of rigid versus flexible models according to case complexity and urgency (Fig. 10). In this scoring system, anatomical features were assigned weighted scores based on their anticipated impact on procedural difficulty: severe angulation (> 60°) was assigned 2 points, while moderate angulation (45°–60°) was assigned 1 point; severe luminal stenosis or true lumen collapse was assigned 2 points; target vessels arising from the aneurysmal sac was assigned 1 point; complex target vessel proximal segment anatomy was assigned 1 point; and severe access tortuosity was assigned 2 points. The total added value score was calculated by summing these individual scores. When the total score was ≥ 2, the flexible model was recommended; when the score was < 2, the rigid model was considered sufficient. In urgent cases (< 48 h), the rigid model was prioritized; in semi-urgent/elective cases (> 48 h), the flexible model was preferred when the score was ≥ 2.

A comparative analysis of fabrication workflow and material properties (Table 5) showed that rigid models were simpler to produce but more expensive, whereas flexible models, although more time-consuming to fabricate, were better suited for simulating dynamic anatomical changes. Overall, rigid models were adequate for standard anatomical evaluation and urgent cases, whereas flexible models provided additional value in cases involving simulation of moderate deformation, hemodynamic simulation and evaluation, and Back-table model trimming.

**Table 5:** Comparison between rigid and flexible 3D-printed models for endovascular simulation

	Rigid model	Flexible model
Fabrication method	Direct printing	Model printing + molding
Fabrication time (with EtO)		
Any models (h)	14.2 ± 1.2	19.3 ± 3.6
TA models (h)	15.0 ± 0.9	21.7 ± 4.1
AA models (h)	13.7 ± 1.1	17.7 ± 2.2
Fabrication cost (without labor)		
Consumable cost per case (USD)	6(AA)/8(TA)	5(AA)/8 (TA)
Initial equipment cost (USD)	1400(PS) + ventilated workspace	800(PS) + 250(PFS)
Material properties		
Color	Transparent	Transparent
Hardness (shore A/D)	85D (rigid)	10–100A (flexible)
Tensile strength (MPa)	48	1.5–45 (adjustable)
Primary applications		
Structural simulation	Yes	Yes
Dynamic simulation	No	Yes
Typical use cases	1. Fenestration positioning 2. Anatomical demonstration and teaching 3. Urgent cases	1.Fenestration positioning 2. Simulation of moderate deformation 3. Hemodynamic simulation and evaluation 4. Back-table model trimming

*EtO* ethylene oxide sterilization, *AD* aortic dissection, *AA* abdominal aorta, *TA* thoracic aorta, *PS* printer system, *PFS* pulsatile flow system

#### Comparison with conventional image-guided group

Compared with the conventional group, the 3DP-guided group demonstrated significantly shorter operative time (133.6 ± 43.3 min vs. 171.4 ± 72.1 min; *p* = 0.039) and reduced contrast volume (181.7 ± 68.6 mL vs. 234.9 ± 101.0 mL; *p* = 0.044). The number of intraoperative device adjustments did not differ significantly between the two groups (0.4 ± 0.9 vs. 0.5 ± 1.1; *p* = 0.643).

The incidence of intraoperative endoleak was 4.5% (1/22) in the 3DP-guided group and 12.5% (3/24) in the conventional group, with no statistically significant difference between the groups (*p* = 0.327). Among these, one case of type Ia endoleak (4.5%) occurred in the 3DP-guided group, while the conventional group had one case of type Ia endoleak (4.2%) and two cases of type II endoleak (8.3%) (*p* > 0.999 and *p* = 0.530, respectively). The incidence of severe complications within 30 days was 4.5% vs. 4.2% (*p* > 0.999) in the two groups, and no postoperative aneurysm rupture or stent migration occurred in either group (Table 6).

**Table 6:** Perioperative outcomes comparison between 3DP-guided group and image-guided group

Parameters	3DP-guided group (*n* = 22)	Image-guided group (*n* = 24)	*p*-value
Operative time (min)	133.6 ± 43.3	171.4 ± 72.1	0.039
Contrast volume used (mL)	181.7 ± 68.6	234.9 ± 101.0	0.044
Number of device adjustments	0.4 ± 0.9	0.5 ± 1.1	0.643
Incidence of intraoperative endoleak (*n* (%))	1 (4.5)	3 (12.5)	0.327
Type Ia	1(4.5)	1 (4.2)	> 0.999
Type II	0(0)	2 (8.3)	0.530
Severe complications within 30 days (*n* (%))	1 (4.5)	1 (4.2)	> 0.999
Aneurysm rupture or stent migration (*n* (%))	0 (0)	0 (0)	-

#### Follow-up comparison with conventional image-guided group

At 1-year follow-up, the all-cause mortality rate was 9.0% (2/22) in the 3DP-guided group and 8.3% (2/24) in the conventional group, with no significant difference between the groups (*p* > 0.999). Target vessel-related complications occurred in one patient (4.2%) in the conventional group, whereas none were observed in the 3DP-guided group (0/22; *p* > 0.999).

The incidence of late endoleak was 13.6% (3/22) in the 3DP-guided group and 16.7% (4/24) in the conventional group (*p* = 0.550). In the 3DP-guided group, late endoleaks included two cases of type II (9.0%) and one case of type IIIa (4.5%). In the conventional group, late endoleaks included one case of type Ia (4.2%), two cases of type II (8.3%), and one case of type IIIb (4.2%). No significant differences were observed in the distribution of endoleak types between the two groups (all *p* > 0.050) (Table 7).

**Table 7:** Follow-up outcomes comparison between 3DP-guided group and image-guided group

Parameters	3DP-guided group (*n* = 22)	Image-guided group (*n* = 24)	*p*-value
All-cause mortality (*n* (%))	2 (9.0)	2 (8.3)	> 0.999
Target vessel-related complications (*n* (%))	0(0)	1 (4.2)	> 0.999
Incidence of Late endoleak (*n* (%))	3 (13.6)	4 (16.7)	0.550
Type Ia	0(0)	1 (4.2)	> 0.999
Type II	2 (9.0)	2 (8.3)	> 0.999
Type IIIa	1 (4.5)	0(0)	0.965
Type IIIb	0 (0)	1 (4.2)	> 0.999

## Discussion

This study yielded four main findings regarding the application of patient-specific static and dynamic 3D-printed models in preoperative planning for endovascular repair of complex aortic disease with physician-modified stent grafts. First, we established a dual-model workflow consisting of a static rigid anatomical model for geometric assessment and fenestration planning, and a dynamic compliant model for device deployment simulation under pulsatile flow conditions. Second, the static models demonstrated good geometric accuracy, and the dynamic models achieved acceptable performance in terms of material mechanical properties and hemodynamic representation. Third, in anatomically complex cases, the dynamic models provided additional planning information beyond that obtainable from static models alone. Fourth, exploratory clinical comparison showed that the 3D-printing-assisted group had shorter operative time and lower contrast volume than the conventional image-guided group, with no significant differences in 1-year follow-up outcomes between the two groups. Taken together, these findings indicate that the patient-specific dual-model strategy is technically feasible and shows potential clinical value in preoperative planning for complex aortic disease.

The core value of the dual-model strategy lies in the complementary division of labor between the two model types. Preoperative planning for complex endovascular aortic repair depends not only on the acquisition of anatomical information but also on the anticipation of device–vessel mechanical interactions. Conventional CTA and workstation-based post-processing remain inherently limited to static morphological information and cannot adequately reflect the post-deployment device configuration under actual anatomical constraints or its dynamic relationship with the aortic wall and target vessels. Within this framework, the static model is used for geometric recognition and fenestration design, whereas the dynamic model is used for visualization of mechanical behavior and decision-making in complex cases. Together, they provide support for different key aspects of preoperative planning.

The static rigid model shifts spatial cognitive tasks traditionally performed intraoperatively to the preoperative stage. In this study, the mean fabrication time for rigid models was 14.2 ± 1.2 h, allowing direct visualization of aortic and branch anatomy at full scale and enabling preoperative determination of fenestration position and orientation. Part comparison analysis showed a mean surface deviation of 0.01 mm, with 97% of surface points deviating by < 0.2 mm, supporting its reliability as a tool for anatomical measurement and fenestration planning. The dynamic compliant model complements the rigid model by providing mechanical information that static models cannot offer—CTA cannot reflect vessel wall compliance, pulsatile deformation, or the mechanical response during stent graft deployment. Material testing showed that silicone 10A most closely approximated native aortic tissue in terms of elastic modulus and tensile strength; dynamic testing demonstrated that peak systolic velocities within the model showed no significant differences from clinical Doppler measurements (all *p* > 0.050), validating the hemodynamic fidelity of the simulation. In cases with severe tortuosity, marked angulation, or short landing zones, these dynamic factors may directly influence stent graft twisting, apposition, and fenestration-to-target vessel alignment. The silicone-based dynamic model combined with a pulsatile flow circuit enabled preoperative simulation of post-deployment configuration and sealing performance. In this study, the flexible model demonstrated distinct advantages in 52.4% (11/21) of cases, primarily in anatomically complex scenarios including severe angulation, dissecting aneurysms with true lumen compression, fenestrations located on the aneurysmal wall, and complex target vessel ostia. Thus, the primary value of the dynamic model lies in providing device-vessel interaction information that static models cannot offer, and this added value is most likely to be realized in patients with complex anatomy and high preoperative uncertainty.

In terms of surgical outcomes, 3D printing-assisted planning was associated with improved procedural efficiency and reduced contrast use. In this study, both operative time and contrast volume were significantly lower in the 3D-printing-guided group than in the conventional group, suggesting that more comprehensive preoperative planning may reduce intraoperative repositioning and additional adjustments. The shorter operative time reflected a smoother intraoperative workflow, while the reduction in contrast volume carried potential renal protective implications. Although the between-group differences in device repositioning and intraoperative endoleak rates did not reach statistical significance, the favorable trends observed in the 3D-printing-guided group suggest that preoperative simulation may help achieve more optimal device positioning at the initial deployment attempt. One-year follow-up showed no significant differences between the two groups in all-cause mortality, target vessel-related complications, or late endoleak rates, indicating that 3D printing-assisted planning was associated with comparable short-term safety to the conventional approach.

In terms of technical feasibility, we systematically evaluated the geometric parameters of the anatomical models and the hemodynamic performance of the dynamic models, with overall consistent results that confirmed the operability of the dual-model workflow. Regarding material selection, the rigid models were directly printed using photopolymer resin, suitable for morphological reproduction and geometric measurement; the dynamic models were fabricated using silicone casting, which balanced elasticity, compliance, and cost-effectiveness, with a wide adjustable hardness range and controllable costs. Although fabrication of the soft models was more complex than that of the rigid ones, the two could be produced concurrently, and the total time was comparable to that required for the dynamic model alone, which was acceptable within the elective surgical timeframe. Building on previous preclinical and educational applications, Coles-Black et al. [15] demonstrated that soft materials better replicate vascular biomechanics, but their work focused primarily on anatomical validation and training, with a reported fabrication time of 24 h and cost of approximately 700 RMB; our fabrication process showed improved efficiency and reduced costs. More recently, Oliny et al. [16]. evaluated soft 3D-printed models in fenestrated EVAR, further highlighting their clinical potential. Building on these foundations, our study provides a direct comparative assessment of rigid and compliant models in complex EVAR planning, with clear evidence of clinical benefit, and offers a practical basis for clinical translation of the dual-model strategy in terms of fabrication accuracy, simulation performance, and resource utilization.

Our findings support a stratified approach to model selection rather than routine application of dynamic models to all cases. Based on our clinical experience, we propose a scoring system-based decision-making algorithm: for cases with standard anatomy and low preoperative uncertainty, rigid models alone may suffice; whereas for complex cases involving severe angulation, marked tortuosity, dissection, or significant compliance-related effects, dynamic models are more valuable. Surgical urgency is also an important consideration—urgent cases (< 48 h) should prioritize rigid models and require assessment of operator availability, and semi-urgent/elective cases (> 48 h) with a score ≥ 2 should preferentially use flexible models. This tailored selection strategy is more aligned with current clinical practice than universal promotion of dynamic simulation.

The potential value of 3D-printed models extends to surgical training, multidisciplinary team communication, and patient education. Rigid models can provide junior surgeons with a tangible understanding of complex anatomy, while dynamic models allow risk-free rehearsal of critical endovascular maneuvers. Physical models may also facilitate team collaboration and help patients and their families better understand the planned intervention. Although these applications were not the primary endpoints of this study, they suggest that the clinical utility of 3D printing may extend beyond its role as a preoperative measurement tool.

## Limitations

This study has several limitations. First, the single-center design and relatively small sample size may limit the generalizability of the results. Second, although the dynamic silicone model enables patient-specific in vitro simulation, it inherently differs from the native aorta regarding perivascular support, internal surface friction, elastic characteristics, and the heterogeneity of material and wall thickness. Specifically, the native vascular intima is covered by an endothelial glycocalyx layer that, when lubricated by blood, yields an ultra-low friction coefficient during device advancement. Furthermore, the homogeneous silicone material cannot fully replicate severe calcification and mural thrombus; consequently, local compliance may be overestimated in calcification-prone regions such as the branch ostia. Looking forward, advancements in perivascular support simulation, multi-material 3D printing, and surface modification technologies, coupled with a reduction in cost barriers, will further enhance the biomechanical realism and practical utility of these models. Concurrently, future large-scale, multicenter studies with long-term follow-up are warranted to validate our findings and further evaluate the feasibility, cost-effectiveness, and broader applicability of this workflow.

## Conclusions

A patient-specific static and dynamic 3D-printing workflow is feasible for preoperative planning of endovascular repair of complex aortic disease with physician-modified stent grafts, with validated geometric accuracy and hemodynamic fidelity. Rigid anatomical models supported anatomic assessment and fenestration planning, whereas compliant dynamic flow models provided additional insight into device deployment in selected anatomically complex cases. In this exploratory study, this workflow was associated with shorter operative time and lower contrast use, with comparable short-term safety to conventional planning.

## Supplementary Information

Supplementary Material 1. Supplementary Movie 1: Procedural simulation workflow based on 3D-printed models.

## Data Availability

The datasets supporting the conclusions of this article are included within the article and its additional files. The data that support the findings of this study are available from the corresponding author upon reasonable request.
